# Factors associated with ADL-defined disability-free survival among patients with advanced cancer in a palliative care setting: a retrospective cohort study

**DOI:** 10.1186/s12904-025-01847-7

**Published:** 2025-07-19

**Authors:** Ryo Soeda, Michiyuki Kawakami, Kengo Nagashima, Tsuyoshi Harada, Takuya Yamaguchi, Yu Furukawa, Tetsuya Tsuji

**Affiliations:** 1 Department of Rehabilitation, Tsurumaki-Onsen Hospital, Hadano, Kanagawa Japan; 2 Department of Rehabilitation Medicine, Keio University Graduate School, Shinjuku, Tokyo Japan; 3 Department of Rehabilitation Medicine, Keio University School of Medicine, Shinjuku, Tokyo, Japan; 4 Biostatistics Unit, Clinical and Translational Research Center, Keio University, Shinjuku, Tokyo, Japan; 5 Department of Rehabilitation Medicine, National Cancer Center Hospital East, Kashiwa, Chiba Japan

**Keywords:** Palliative care unit, Disability-free survival, Advanced cancer, Eating, Toileting, Walking

## Abstract

**Objectives:**

Disability-free survival (DFS) focusing on essential activities of daily living (ADL) is a critical outcome for patients with advanced cancer receiving palliative care, yet remains underexplored. This study aimed to examine DFS for core ADL components (eating, toileting, and walking) in patients admitted to the palliative care unit (PCU) and to identify associated factors using a competing risk model. Understanding these factors may guide targeted interventions to preserve functional independence and enhance quality of life.

**Methods:**

This retrospective cohort study included advanced cancer patients admitted to the PCU between August 2018 and September 2022, excluding those discharged home. The primary endpoint was DFS, defined as the period from admission to an event resulting in a Functional Independence Measure (FIM™) score below 6 in eating, toileting, or walking. FIM™ is a widely used tool for assessing functional independence across multiple domains. DFS was estimated using a competing risk model to account for death as a competing event, and Fine–Gray regression analysis was conducted to identify factors associated with DFS.

**Results:**

A total of 143, 48, and 46 patients were analyzed for eating, toileting, and walking, respectively. The median DFS (95% confidence interval) was 4 (3–5) weeks for eating, 3.5 (3–6) weeks for toileting, and 3 (2–6) weeks for walking. Cognitive function (FIM™ cognitive items) and the cachexia (modified Glasgow Prognostic Score) at admission were commonly associated with DFS. Neutrophil-to-lymphocyte ratio was specifically associated with DFS for eating, and bone and liver metastases were associated with DFS for toileting and walking.

**Conclusions:**

Monitoring ADL-defined DFS and key factors such as cognitive function and inflammation-nutritional status may enable timely interventions, optimize care strategies, and support quality of life for patients with advanced cancer in palliative care settings. Future studies could explore prospective validation of these findings and investigate whether early interventions targeting these factors can extend disability-free survival. In addition, integrating rehabilitation strategies and routine cognitive assessments may further enhance the benefits of individualized care in this population.

**Supplementary Information:**

The online version contains supplementary material available at 10.1186/s12904-025-01847-7.

## Introduction

Disability in activities of daily living (ADL) decreases a patient’s quality of life (QOL) as these activities are performed repeatedly throughout the day. A previous study reported that approximately one in three adult cancer patients experiences ADL disability [1], highlighting a significant challenge in clinical management and palliative care. Previous studies have shown that older cancer patients rate ADL maintenance as a top priority [2], and that declines in ADL can lead to existential distress and feelings of being a burden to others [3, 4]. Consequently, interventions to prevent or mitigate ADL deterioration are considered crucial for improving patient outcomes.

A good death is considered a time when a person can stay as independent as possible, experience little pain, and maintain close ties with loved ones [5]. Patients who maintain their ADL are better prepared for end of life and feel that their dignity is protected [6]. Once function starts to decline, they often feel cut off from themselves and lose their sense of control [7]. Heavy dependence increases the feeling of being a burden [8]. Thus, a decline in ADL is not only a physical change, but also an existential turning point. Measuring the time each ADL stays intact—its disability-free survival (DFS)—is therefore important for planning care that supports self-worth and helps patients achieve a good death.

The concept of DFS has recently become a topic of discussion, as it captures the period during which patients maintain functional independence—an aspect that is not fully reflected by overall survival alone. DFS has been studied in various medical fields, including cancer [9–11], heart disease [12], diabetes [13], older age [14], and chronic kidney disease [15]. However, in advanced cancer patients, especially those receiving palliative care, ADL-defined DFS remains unclear. Among ADLs, previous work suggests that toileting, eating, and walking (mobility) are particularly important for cancer patients [2]. Guided by this patient-centered hierarchy, we chose to evaluate DFS separately for these three items. We used the Functional Independence Measure (FIM) [16, 17], an 18-item instrument scored on a 7-point scale—is widely recognized as a comprehensive tool for evaluating the graded levels of functional independence.

Building on these findings, we focused on these three key ADL items [2] and considered the competing risks inherent in advanced cancer (e.g., cancer-related mortality). In cancer patients, eating is generally the easiest ADL to maintain, whereas toileting and walking tend to lose independence much earlier [18]. Because the difficulty of each FIM™ item varies markedly [19, 20], analyzing all patients in a single dataset would mix individuals with very different functional thresholds and obscure item-specific trajectories. We therefore created three independent baseline datasets, each limited to patients who were fully independent in the target item at admission, and calculated item-specific DFS for toileting, eating and walking. This design aligns with patients’ stated priorities, respects inter-item heterogeneity, and yields clinically relevant time frames for anticipatory rehabilitation and care planning. This retrospective cohort study aimed to identify the patient characteristics and clinical factors associated with ADL-defined DFS among patients with advanced cancer admitted to a palliative care unit (PCU), and thus help guide care and rehabilitation strategies to support their daily lives, improve QOL, and facilitate better discharge planning in PCUs.

## Methods

### Materials and methods

This retrospective cohort study was conducted using electronic medical records and the Rehabilitation Department’s medical database at Tsurumaki-Onsen Hospital in Kanagawa, Japan. The study participants were patients with cancer who had been discharged from the PCU of Tsurumaki-Onsen Hospital between August 1, 2018, and September 30, 2022. Admission to the PCU is restricted to patients with advanced, life-limiting cancer who require intensive symptom management and multidisciplinary end-of-life care, including relief of refractory pain, dyspnea, and other distressing symptoms. Importantly, the case mix of Japanese PCUs differs from that of palliative-care wards in many other countries: Japanese units have a mean length of stay (LOS) of ~ 28 days, in-unit death is over 70% (all patients in the present cohort died on the ward), and nationwide data show that only about 12% of admissions are discharged home [21]. By contrast, North-American Acute PCUs are short-stay, discharge-oriented facilities, with a typical LOS of ~ 10 days, an in-unit death rate below 50%, and approximately one-third of patients returning home after rapid symptom control [22]. These structural differences imply divergent patient characteristics and must be kept in mind when interpreting disability-free survival across settings. The eligibility criteria were as follows: (1) patients diagnosed with advanced/metastatic cancer, (2) patients who were hospitalized in the PCU, (3) age > 18 years, (4) patients with more than one ADL assessment, and (5) patients with an FIM™ score of 6 or 7 at admission for the eating, toileting, and walking items (see scoring criteria below). Patients with missing ADL assessments or incomplete inpatient laboratory data were excluded. In addition, patients discharged to home were excluded because they were generally planned for home discharge and were not expected to have the same DFS as those who died.

ADL assessment was performed using the Japanese version of the FIM™ version 3.0 [16, 17]. The FIM™ evaluates ADL 18 items on a 7-point scale, ranging from 1 (full assistance) to 7 (independent). It includes 13 motor items and five cognitive items. The total score ranges from 18 to 126, with higher values indicating greater independence. FIM™ scores of 6 (modified independent) and 7 (independent) indicate that the patient is considered independent for that item, whereas scores of 5 (supervised) or below indicate that assistance is required. FIM™ was evaluated every 1 or 2 weeks after admission to the PCU by a physical therapist, occupational therapist, speech-language therapist, or nurse trained in the evaluation method. Each patient received a rehabilitation prescription from a physician, and interventions were provided by physical therapists, occupational therapists, or speech-language pathologists with the aim of improving their QOL.

This study was conducted according to the Declaration of Helsinki. Approval was obtained from the Tsurumaki-Onsen Hospital Clinical Research Ethics Review Subcommittee (approval No. 515). Eligible patients or their family members signed a comprehensive consent form upon admission to provide data for clinical research. Additional information and consent were not required for this study.

### Data collection

The following information was collected from patients’ medical records: age, sex, diagnosis (primary cancer site), presence of metastasis (e.g., brain, bone, liver, lung), and duration of stay in the PCU. The primary endpoint was DFS, defined as the period from admission to the PCU to the occurrence of an event resulting in a decrease in the score of any of the eating, toileting, or walking items of the FIM™ below 6 points (assisted) for each individual dataset.

Data collected on admission were modified Glasgow prognostic score (mGPS) [23], neutrophil-to-lymphocyte ratio (NLR) [24], and prognostic nutritional index (PNI) [25, 26]. mGPS and NLR are indicators of systemic inflammation. The mGPS classifies patients into three levels based on albumin and CRP levels, as follows: CRP < 10 mg/L = 0; CRP > 10 mg/L = 1, CRP > 10 mg/L and albumin < 35 g/L = 2 [23]. NLR was defined as absolute neutrophil count divided by absolute lymphocyte count [24]. PNI was calculated using the following formula: 10 × albumin g/dL + 0.5% × total lymphocyte count [26].

### Data analysis and statistics

Separate datasets were created for patients who were independent in eating, toileting, and walking. Descriptive statistics were conducted first to analyze the demographic attributes of the subjects. The three groups were then compared in terms of the eating, toileting, and walking items from the FIM™ scale. For these comparisons, Pearson’s chi-square test or Fisher’s exact test was used for nominal measures, and the Kruskal–Wallis test was conducted for all continuous variables without normal distribution. When significant differences were found among the three groups, multiple comparisons were performed using the Bonferroni method.

The DFS period was calculated as the cumulative event rate using a competing risk model until the FIM™ score was less than 6 points (indicating the need for assistance). Mortality events were considered competing risks, and discharge from the PCU (hospital or facility) was considered censored data. To identify factors associated with DFS, Fine–Gray regression analysis was conducted using the occurrence of events needing assistance as the objective variable and age, sex, presence of metastasis, FIM ^TM^ cognitive items, mGPS, NLR, and PNI as explanatory variables. The cumulative incidence of competing risks between two groups was also compared, using Gray’s test. The statistical significance threshold was set at 5%. Statistical analysis was performed using EZR on R commander ver. 1.55 (The R Foundation for Statistical Computing, Vienna, Austria) [27].

## Results

### Participant demographics

**Fig. 1:**
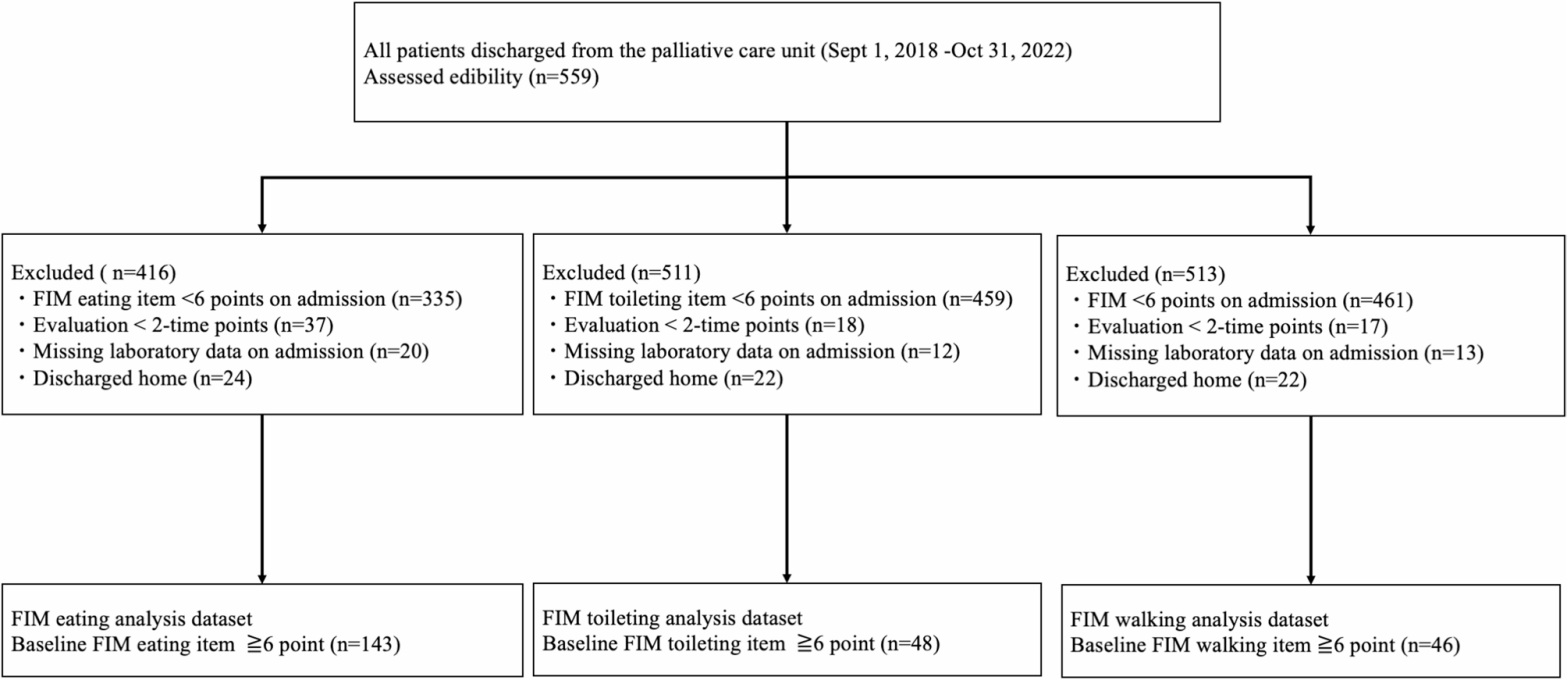
Flowchart of patient inclusion

A total of 559 patients were discharged during the study period. After applying the inclusion and exclusion criteria, 143 patients were eligible for the eating item analysis, 48 for the toileting item analysis, and 46 for the walking item analysis (Fig. 1). The subjects’ demographics are shown in Table 1. The median age was 77 years for eating, 74.5 years for toileting, and 74.5 years for walking items. Brain and bone metastases were more frequent; and FIM ^TM^ motor items, FIM ^TM^ cognitive items, and FIM ^TM^ total scores were significantly lower in the eating item group than in the toilet item and walking item groups.

**Table 1:** Participant characteristics

	Eating item (*n* = 143)	Toileting item (*n* = 48)	Walking item (*n* = 46)	Comparison among three groups	Comparison between two groups*
Sex					
female	82 (57.3)	25 (52.1)	27 (58.7)	0.774§	
male	61 (42.7)	23 (47.9)	19 (41.3)		
Age (years)	77.00 [69.00, 85.00]	74.50 [67.75, 79.25]	74.50 [67.25, 80.75]	0.139#	
Cancer type				0.938†	
Upper gastrointestinal	46 (32.2)	13 (27.1)	15 (32.6)		
Lower gastrointestinal	16 (11.2)	3 (6.2)	3 (6.5)		
Thoracic	28 (19.6)	12 (25.0)	9 (19.6)		
Breast	13 (9.1)	4 (8.3)	4 (8.7)		
Gynecological	8 (5.6)	6 (12.5)	5 (10.8)		
Genitourinary	8 (5.6)	2 (4.2)	2 (4.3)		
Head and neck	4 (2.8)	0 (0.0)	0 (0.0)		
Skin/soft tissue	4 (2.4)	2 (4.2)	2 (4.4)		
Other	16 (11.2)	6 (12.5)	6 (13.2)		
Metastasis					
brain	16 (11.2)	0 (0.0)	0 (0.0)	0.004§	a, b
bone	31 (21.7)	3 (6.2)	4 (8.7)	0.013§	a
lung	43 (30.1)	13 (27.1)	13 (28.3)	0.916§	
liver	45 (31.5)	15 (31.2)	16 (34.8)	0.908§	
Admission source					
Home	75 (52.4)	26 (54.2)	28 (60.9)	0.947§	
Facility as home	10 (7.0)	2 (4.2)	2 (4.3)		
Another ward	1 (0.7)	0 (0.0)	0 (0.0)		
Long-term care hospital	1 (0.7)	0 (0.0)	0 (0.0)		
Acute care hospital	56 (39.2)	20 (41.7)	16 (34.8)		
Hospitalization	48.00 [26.50, 86.50]	49.50 [28.75, 89.25]	46.00 [28.50, 87.50]	0.857#	
Albumin (g/dL)	3.00 [2.50, 3.50]	3.30 [2.68, 3.52]	3.30 [2.62, 3.50]	0.192#	
CRP (mg/dL)	2.54 [0.92, 6.62]	2.56 [0.51, 6.62]	2.56 [0.58, 10.77]	0.902#	
mGPS					
0	39 (27.3)	15 (38.5)	16 (34.8)	0.668§	
1	19 (13.3)	5 (12.8)	6 (13.0)		
2	85 (59.4)	19 (48.7)	24 (52.2)		
Lymphocyte actual count	884.00 [665.55, 1262.75]	875.80 [631.30, 1313.85]	843.15 [619.80, 1205.92]	0.833#	
Neutrophil actual count	5435.20 [3692.15, 7873.30]	5548.60 [3606.03, 8300.85]	5432.00 [3585.90, 7972.20]	0.909#	
PNI	35.23 [28.46, 40.38]	37.18 [30.73, 41.35]	37.18 [30.25, 41.28]	0.216#	
NLR	6.18 [3.48, 11.01]	6.15 [3.27, 13.42]	6.17 [3.05, 14.10]	0.999#	
FIM motor score	56.00 [41.00, 69.50]	78.00 [68.00, 82.25]	77.00 [67.00, 82.00]	< 0.001#	a, b
FIM cognitive score	30.00 [26.00, 35.00]	35.00 [31.00, 35.00]	35.00 [32.25, 35.00]	< 0.001#	a, b
FIM total score	83.00 [66.00, 102.50]	111.00 [101.75, 116.25]	108.50 [99.50, 116.75]	< 0.001#	a, b

Values are n (%) or median [25%, 75%]. Metastases are duplicate subjects. CRP: C-reactive protein, mGPS: modified Glasgow Prognostic Score, PNI: prognosis nutritional index, NLR: neutrophil-to-lymphocyte ratio FIM: functional independence measure. #Comparison using the Kruskal–Wallis Test, †comparison using the chi-square test, §comparison using the Fisher exact test, *p < 0.05 for 2-group comparison with eating item; a: toileting item, b: walking item, with Bonferroni correction (no significance were observed in toileting and walking)

Compared with the analyzed cohort, patients discharged home tended to be younger, were more frequently diagnosed with breast cancer and bone metastasis and were admitted from their own residence　(Supplemental Material 1). They generally showed better nutritional status and lower systemic-inflammation indices. FIM ^TM^ scores for toileting and walking were comparable between the two groups.

### Disability-free survival period and related factors

**Fig. 2:**
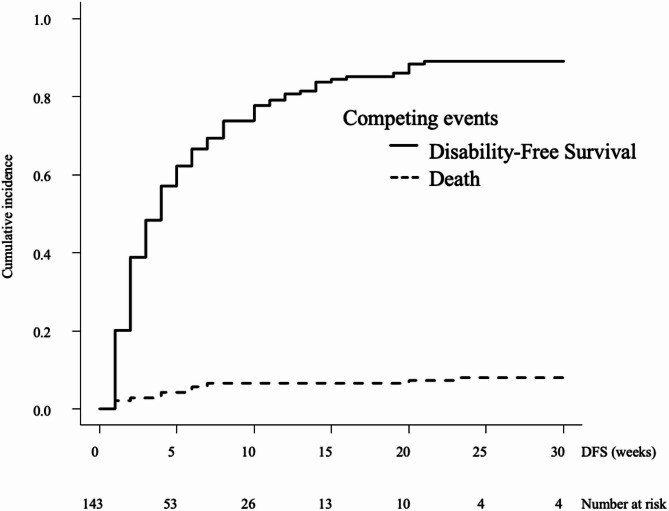
Disability-free survival for the eating item. The cumulative incidence of events is shown, including conflicting events

**Fig. 3:**
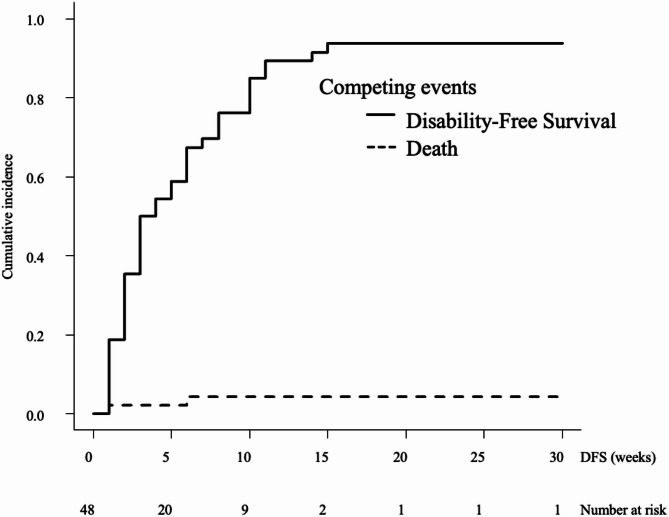
Disability-free survival for the toileting item. The cumulative incidence of events is shown, including conflicting events

**Fig. 4:**
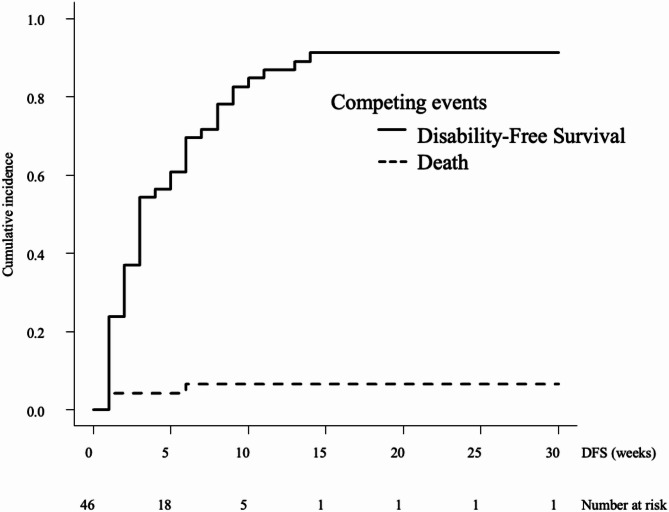
Disability-free survival for the walking item. The cumulative incidence of events is shown, including conflicting events

The median DFS time after admission for the eating item was 4 weeks (95% confidence interval [CI]: 3–5) (Fig. 2). Associated factors were FIM™ cognitive items (hazard ratio [HR]: 0.96, 95%CI: 0.94–0.99, *p* = 0.006), mGPS (HR: 1.21, 95%CI: 1.01–1.44, *p* = 0.037), and NLR (HR: 1.02, 95%CI: 1.01–1.04, *p* = 0.003) (Table 2). The median DFS time after admission for the toileting item was 3.5 weeks (95%CI: 3–6) (Fig. 3). Associated factors were bone metastasis (HR: 4.31, 95%CI: 2.18–8.50, *p* < 0.001), liver metastasis (HR: 1.84, 95%CI: 1.09–3.10, *p* = 0.022), FIM™ cognitive items (HR: 0.92, 95%CI: 0.87–0.98, *p* < 0.001), and mGPS (HR: 1.44, 95%CI: 1.02–2.03, *p* = 0.035) (Table 2). The median DFS time after admission for the walking item was 3 weeks (95%CI: 2–6) (Fig. 4). Associated factors were bone metastasis (HR: 2.36, 95%CI: 1.05–5.28, *p* = 0.037), liver metastasis (HR: 1.86, 95%CI: 1.07–3.25, *p* = 0.028), FIM™ cognitive items (HR: 0.92, 95%CI: 0.87–0.97, *p* < 0.001), and mGPS (HR:1.48, 95%CI: 1.06–2.07, *p* = 0.023) (Table 2).

**Table 2:** Factors related to disability-free survival

Factor	Eating			Toileting			Walking	
	HR (95%CI)	*p* value		HR (95%CI)	*p* value		HR (95%CI)	*p* value
Male	0.85 (0.61–1.18)	0.34		0.67 (0.36–1.27)	0.22		0.60 (0.34–1.05)	0.076
Age	1.01 (1–1.02)	0.17		1.01 (0.99–1.04)	0.22		1.02 (0.99–1.04)	0.22
Brain metastasis	1.47 (0.76–2.84)	0.26		NA	NA		NA	NA
Bone metastasis	0.95 (0.61–1.47)	0.81		4.31 (2.18–8.50)	< 0.001		2.36 (1.05–5.28)	0.037
Lung metastasis	0.88 (0.60–1.28)	0.50		0.78 (0.38–1.60)	0.50		0.55 (0.26–1.14)	0.11
Liver metastasis	1.36 (0.98–1.88)	0.063		1.84 (1.09–3.10)	0.022		1.86 (1.07–3.25)	0.028
FIM cognitive score	0.96 (0.94–0.99)	0.006		0.92 (0.87–0.98)	< 0.001		0.92 (0.87–0.97)	< 0.001
mGPS	1.21 (1.01–1.44)	0.037		1.44 (1.02–2.03)	0.035		1.48 (1.06–2.07)	0.023
PNI	0.99 (0.97–1.02)	0.69		0.98 (0.94–1.02)	0.27		0.97 (0.93–1.01)	0.10
NLR	1.02 (1.01–1.04)	0.003		1.02 (0.98–1.06)	0.36		1.02 (0.99–1.05)	0.24

Brain metastasis is not shown for toileting and walking as there was only one patient with brain metastases. HR: hazard ratio, CI: confidence interval, FIM: functional independence measure, mGPS: modified Glasgow prognostic score, PNI: prognosis nutritional index, NLR: neutrophil-to-lymphocyte ratio, NA (not available) indicates that no patients had brain metastasis; therefore, this variable was not included in the model

**Fig. 5:**
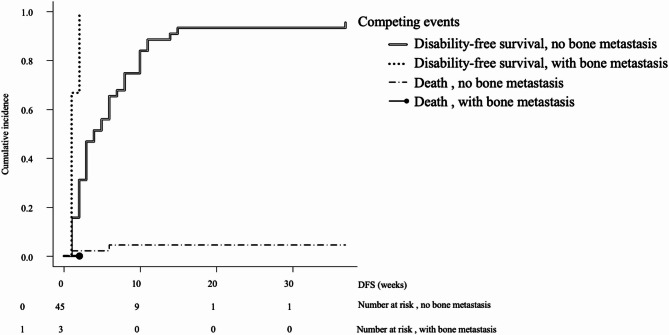
Disability-free survival for the toileting item according to the occurrence of bone metastasis. The cumulative incidence of events is shown, including conflicting events

DFS was 1 week (95% CI: 1–2 weeks) and 4 weeks (95% CI: 3–7 weeks) for patients with and without bone metastases, respectively (statistically significant difference, *p* = 0.001) (Fig. 5).

## Discussion

This study is the first to investigate DFS and the factors related to DFS in advanced cancer patients admitted to a PCU. We found that DFS after admission was 4 weeks for the eating item, 3.5 weeks for the toileting item, and 3 weeks for the walking item. Of note, cognitive function (assessed by FIM™ cognitive items) and cachexia (assessed by mGPS) at admission emerged as significant predictors across all three ADL domains.

There are limited reports regarding ADL after admission to a PCU. Hiratsuka et al. identified factors associated with declining abilities in walking, eating, and communicating 2 weeks (14 days) after admission to a PCU, but did not report DFS [28]. In their study, each ADL was defined as Eastern Cooperative Oncology Group Performance Status 3 or less for walking, ability to eat more than a few meals, and Communication Capacity Scale 2 or more for communication. Because these definitions do not clearly delineate whether a patient is fully independent or requires assistance in daily living, their applicability to detailed care and rehabilitation planning is limited.

By defining DFS, this study is the first to show that ADL independence after admission to a PCU lasts only about 1 month before assistance is needed. This study demonstrates the value of tailoring care plans to ADL-defined DFS. Furthermore, continuous monitoring of critical factors such as cachexia, cognitive decline, and metastases from admission may guide earlier, individualized interventions to refine discharge planning and support patients’ independence both at home and during hospitalization.

Cognitive function (FIM™ cognitive items) and cachexia (mGPS) were identified as common factors associated with all three items. Cognitive impairment in patients with cancer has been associated with ADL disability [29] and has been reported to increase the risk of falls [30]. Cognitive function is necessary for safe performance of activities and is important for patients with advanced cancer, whose physical functions are likely to decline. The mGPS serves as an indicator of cachexia by assessing albumin and CRP. Patients with cachexia are known to have weak muscle strength and poor toileting and walking performance [31–33]. Thus, cachexia likely contributed to a shorter DFS by accelerating muscle loss and reducing functional performance.

NLR was a significant factor in the eating item. Patients in the eating item group had a lower FIM™ at admission and their ADLs had already declined. Systemic inflammation (NLR) may contribute to further ADL deterioration. The cessation of oral intake indicates that the end of life is approaching [34]; however, the lack of significant differences in duration of hospitalization among the three item groups suggests that the frequency of eating is not the only factor determining life prognosis. The present findings underscore the importance of systemic inflammation as an indicator for further ADL decline.

The toileting and walking items were commonly associated with the factors of bone and liver metastasis. Bone metastasis causes skeletal-related events and leads to a decline in ADL and predominantly affects the spine and femur. Metastasis to the spine and pelvis significantly impacts the maintenance of lower limb function, making it a crucial factor in the decline of ADL [35, 36]. Bone metastases were associated with the highest hazard ratio for toileting dependency, an ADL that patients rate as most important. Consequently, DFS for toileting was significantly shorter in patients with bone metastases, indicating a need for tailored rehabilitation and care strategies to maintain QOL and prolong ADL-defined DFS. Cirrhosis demonstrates how liver problems can harm basic ADLs in several ways [37]. Muscle loss and poor nutrition weaken the legs and trunk, and ascites and leg swelling cause joint stiffness. Hepatic encephalopathy makes it harder to plan and carry out movements. Faster aging and more depressive moods can further reduce energy and focus. Together, these changes make toileting, transferring, and dressing difficult. This pattern was seen in patients waiting for a liver transplant, where these ADLs were most often impaired [37]. We believe that liver metastases cause a similar chain of problems. Fluid build-up, muscle wasting, and cognitive changes from encephalopathy reduce functional ability. As a result, patients lose independence sooner, which may help explain the shorter DFS for toileting and walking observed in the present study.

This study has several limitations that warrant careful consideration. First, it was conducted at a single center with an older cohort and an uneven distribution of primary cancer sites; therefore, external validity is limited and DFS should be confirmed in multicenter cohorts with more diverse demographic and oncologic characteristics. Second, key explanatory variables—such as rehabilitation intensity and frequency, palliative medications (e.g., opioids, benzodiazepines), and systematic symptom measures (pain, fatigue, delirium scores)—were not captured in the retrospective dataset. Because these factors plausibly influence functional trajectories, future prospective studies should incorporate detailed information on rehabilitation interventions, pharmacologic and non-pharmacologic palliative care, and serial symptom outcomes to refine risk stratification models. Finally, all participants were inpatients who received physician-prescribed rehabilitation, which may have mitigated functional decline and thereby limited the generalizability of the findings to settings where rehabilitation services are less available or delivered differently.

Despite these limitations, this study identified DFS and its associated factors in advanced cancer patients admitted to a PCU. The item-specific DFS values and their determinants underscore the necessity of assessing ADL status, cachexia, cognitive function, systemic inflammation, and metastatic burden at admission—even in patients who appear fully independent. Three patient-centred applications emerge from these data. First, cognitive impairment and a high mGPS provide a simple admission screen that pinpoints individuals at high risk of rapid functional decline, thereby guiding the preferential allocation of rehabilitation resources and early discharge planning. Second, because loss of ADL independence is linked to existential distress [3, 4], flagging this high-risk group creates an opportunity for timely referral to psychological and spiritual-care services, helping patients maintain dignity at the end of life. Third, the DFS timeline—approximately three weeks for walking and toileting and four weeks for eating—offers a concrete schedule for intervention: intensive PT/OT can be front-loaded within the first two weeks, while occupation-based eating support and nutritional counselling can be introduced during weeks 2–3. Collectively, these insights provide benchmarks for phased palliative-care planning; by anticipating functional decline, the care team can enhance quality of life and improve the overall end-of-life experience for patients and their families.

## Conclusion

DFS for cancer patients after admission to the PCU was 4 weeks for eating, 3.5 weeks for toileting, and 3 weeks for walking. Factors common to all three activities were cognitive function (FIM™ cognitive items) and cachexia (mGPS). These DFS data suggest that by appropriately assessing patients who remain ADL-independent at admission and adopting a phased approach—anticipating the period of preserved autonomy and planning the subsequent interventions—clinicians may alleviate the physical and psychological distress associated with ADL decline.

## Electronic Supplementary Material

Below is the link to the electronic supplementary material.

Supplemental Material 1

## Data Availability

Data availabilityThe datasets generated and/or analyzed during the present study are available from the corresponding author upon reasonable request.

## Abbreviations

ADL: Activities of daily living
QOL: Quality of Life
DFS: Disability-free survival
FIM: Functional Independence Measure
mGPS: Modified Glasgow prognostic score
NLR: Neutrophil-to-lymphocyte ratio
PNI: Prognostic nutritional index
