# Supplementary material for: Factors associated with ADL-defined disability-free survival among patients with advanced cancer in a palliative care setting: a retrospective cohort study
Source: BMC Palliat Care. 2025 Jul 19;24:206. doi: 10.1186/s12904-025-01847-7 (PMC12275397; doi:10.1186/s12904-025-01847-7)
Supplement: Supplementary file 1 — Supplemental Material 1 [file 12904_2025_1847_MOESM1_ESM.docx]

**Supplemental Table. Characteristics of Patients Discharged Home**

|  | Eating item (n=24) | Toileting item (n=22) | Walking item (n=22) |  |  |
| --- | --- | --- | --- | --- | --- |
| Sex |  |  |  |  |  |
| female | 17 (70.8) | 15 (68.2) | 15 (68.2) |  |  |
| male | 7 (29.2) | 7 (31.8) | 7 (31.8) |  |  |
| Age (years) | 67.00 [66.00, 79.25] | 67.00 [66.00, 73.00] | 67.00 [66.00, 73.00] |  |  |
| Cancer type |  |  |  |  |  |
| Upper gastrointestinal | 1 (4.2) | 1 (4.5) | 1 (4.5) |  |  |
| Lower gastrointestinal | 2 (8.3) | 1 (4.5) | 1 (4.5) |  |  |
| Thoracic | 3 (12.5) | 2 (9.1) | 2 (9.1) |  |  |
| Breast | 13 (54.2) | 13 (59.1) | 13 (59.1) |  |  |
| Genitourinary | 1 (4.2) | 0 (0.0) | 0 (0.0) |  |  |
| Head and neck | 1 (4.2) | 4 (18.2) | 4 (18.2) |  |  |
| Other | 2 (8.3) | 0 (0.0) | 1 (4.5) |  |  |
| Metastasis |  |  |  |  |  |
| brain | 1 (4.2) | 1 (4.5) | 1 (4.5) |  |  |
| bone | 16 (66.7) | 14 (63.6) | 15 (68.2) |  |  |
| lung | 5 (20.8) | 3 (13.6) | 3 (13.6) |  |  |
| liver | 12 (50.0) | 11 (50.0) | 12 (54.5) |  |  |
| Admission source |  |  |  |  |  |
| Home | 24 (100.0) | 22 (100.0) | 22 (100.0) |  |  |
| Hospitalization | 16.50 [14.00, 25.00] | 17.50 [14.25, 25.00] | 17.50 [14.25, 27.25] |  |  |
| Albumin (g/dL) | 3.75 [3.58, 4.00] | 3.90 [3.70, 4.20] | 3.85 [3.70, 4.20] |  |  |
| CRP (mg/dL) | 0.16 [0.09, 0.88] | 0.10 [0.07, 0.18] | 0.10 [0.07, 0.18] |  |  |
| mGPS |  |  |  |  |  |
| 0 | 18 (75.0) | 18 (81.8) | 18 (81.8) |  |  |
| 1 | 2 (8.3) | 2 (9.1) | 2 (9.1) |  |  |
| 2 | 4 (16.7) | 2 (9.1) | 2 (9.1) |  |  |
| Lymphocyte actual count | 821.40 [716.90, 1212.07] | 815.55 [725.90, 1101.17] | 815.55 [725.90, 965.73] |  |  |
| Neutrophil actual count | 2964.60 [2071.27, 4927.95] | 2964.60 [2062.42, 6819.75] | 2964.60 [2062.42, 6819.75] |  |  |
| PNI | 41.61 [40.19, 43.80] | 42.69 [40.83, 48.82] | 42.33 [40.77, 48.65] |  |  |
| NLR | 3.18 [2.36, 5.71] | 3.18 [2.50, 7.53] | 3.50 [2.58, 7.53] |  |  |
| FIM motor score | 72.00 [71.00, 76.25] | 72.00 [72.00, 78.75] | 72.00 [72.00, 74.75] |  |  |
| FIM cognitive score | 35.00 [29.75, 35.00] | 35.00 [31.50, 35.00] | 35.00 [31.50, 35.00] |  |  |
| FIM total score | 107.00 [99.00, 108.75] | 107.00 [104.00, 112.75] | 107.00 [104.00, 108.50] |  |  |
| Values are n (%) or median [25%, 75%]. Metastases are duplicate subjects. CRP: C-reactive protein, mGPS: modified Glasgow Prognostic Score, PNI: prognosis nutritional index, NLR: neutrophil-to-lymphocyte ratio FIM: functional independence measure. #Comparison using the Kruskal–Wallis Test, †comparison using the chi-square test, §comparison using the Fisher exact test, *p<0.05 for 2-group comparison with eating item; a: toileting item, b: walking item, with Bonferroni correction (no significance were observed in toileting and walking). | | | | |  |
|  |  |  |  |  |  |
|  |  |  |  |  |  |
|  |  |  |  |  |  |
